# Development of a categorical naming test in Korean: Standardization and clinical application for patients with stroke

**DOI:** 10.1371/journal.pone.0247118

**Published:** 2021-02-19

**Authors:** Yu Mi Hwang, Yoonhye Na, Sung-Bom Pyun

**Affiliations:** 1 Brain Convergence Research Center, Korea University College of Medicine, Seoul, Republic of Korea; 2 Department of Biomedical Sciences, Korea University College of Medicine, Seoul, Republic of Korea; 3 Department of Physical Medicine and Rehabilitation, Korea University College of Medicine, Seoul, Republic of Korea; University of Central Florida, UNITED STATES

## Abstract

**Purpose:**

The purpose of this study was to develop and standardize a new categorical naming test, titled the Categorical Naming Test (CNT), for stroke patients, and to investigate its validity and clinical usefulness for patients with stroke.

**Materials and methods:**

The CNT was developed based on semantic category, imageability, and psycholinguistic factors such as word frequency and word length. The test materials included two main semantic categories (living objects and artificial objects) comprising 60 items. We standardized the CNT on 221 healthy adults and administered the CNT to 112 stroke patients.

**Results:**

Internal consistency and concurrent validity of the test were high. The mean total CNT scores varied significantly according to participants’ age, sex, and education. Among healthy controls, the scores for naming living objects were significantly higher than those for artificial objects. The analysis of stroke patients showed that the total CNT score revealed a statistically significant difference based on the patients’ lesion laterality and presence of aphasia, after controlling for age, sex, and education. However, the categorical scores achieved by comparing the naming scores for living and artificial objects showed no significant differences according to lesion laterality, stroke type, and presence of aphasia.

**Conclusion:**

The CNT is a newly developed version of an overt naming task with high internal consistency validity for stroke patients in Korea. The newly developed CNT can prove useful in evaluating naming ability in stroke patients.

## Introduction

Difficulty in finding words is one of the most common features of language impairment among individuals with aphasia after stroke [1–4]. It is characterized by problems associated with recall of words, names in particular, and reflects a primary language disorder or other non-linguistic cognitive deficits [5, 6].

To identify the factors underlying these naming difficulties, a number of theories about the picture naming process have been proposed, and previous research has shown that complex mechanisms are involved in picture naming. Among these, the psycholinguistic model explains the related cognitive functions.

The picture naming process entails a number of relatively distinct but interacting mental representations and cognitive processes such as: (1) recognition of the visual stimulus as a familiar concept, (2) accessing the meaning of the object, (3) accessing the phonological word form, and (4) motor programming, planning articulation, and implementation of the movement sequences to utter the word. Although these functions may not be completely anatomically isolated, they can be individually impaired following brain damage. Therefore, they are depicted as distinct levels of processes underlying naming [7–10]. Naming deficits can be the result of a multi-faceted operation involved in each processing level or an impairment between processing levels, as naming requires an intricate interplay between various cognitive processes.

Several clinical studies have reported various cases of naming deficit; in particular, the category-specific deficit was shown in patients with a wide variety of neurological pathologies including stroke, herpes simplex virus encephalitis, traumatic brain injury, temporal lobe epilepsy, and other conditions [11–15]. Category-specific deficits originated from studies of neurological patients who showed selective impairment in recognizing particular word classes of objects. Since Warrington’s publications in the 1970s and early 1980s [16, 17], several studies have reported category-specific impairment, neural substrates of mental lexicon, and conceptual knowledge associated with the naming process. The common dissociation reported in these patients was a selective naming impairment involving living objects relative to artificial objects [18–21]. An opposite type of dissociation associated with naming artificial objects has also been reported [20]. Therefore, it is very important that naming tasks adequately sample performance across an appropriate number of semantic categories. In order to detect selective semantic impairment, tests of confrontation naming should include items from various subcategories which have been reported to reveal dissociation in naming different categories of items.

Previous literature investigating language disorders has reported cognitive and psycholinguistic mechanisms underlying language processing. Visual confrontation naming tests (e.g., picture naming test) are widely used in clinical settings, and they play an important role in understanding human cognition and its underlying neurological mechanisms [4]. Various tests have been developed to assess visual confrontation naming. They are diverse in terms of the number of items, number of different word categories, methods of item presentation (items presented in the order of estimated difficulty or items presented in a random order), visual format of stimulus items (colored photographs, grayscale drawings, and black-and-white line drawings), and application of the test to different participant groups (under monolingual, bilingual, or multilingual circumstances) [22–27]. The Boston Naming Test (BNT) [22] is probably the most widely used test to assess visual confrontation naming, and it has been adapted in a variety of languages. However, the BNT omits some word categories such as insects, body parts, and fruits; it only has one exemplar in the categories of bird (“pelican”), flower (“flower”), and vegetable (“asparagus”) and does not adequately sample the range of possible semantic categories required to identify selective semantic impairment. Moreover, item difficulty in the BNT increases with item progression and the latter part of the test is mostly composed of artificial objects. Other tests such as the NAB Naming Test [23] have excellent norms and good design for re-testing, but do not allow for dissociation between different word categories of items used in the test.

Many studies have emphasized the importance of normative data, which plays a substantial role in word retrieval by patients with aphasia and other types of brain damage. Although many standardized tests have been developed, specialized naming tests to identify the selective semantic impairments are still rare. In this context, there is a need for an in-depth test with a balance between the number of items for living and artificial objects, and an evenly distributed number of items within each semantic category according to word frequency where word length is controlled for. The newly developed CNT in this study is an in-depth test to investigate naming performance for two categories, that is, living and artificial objects, in more detail. The aim of the present study was to develop and standardize a new categorical naming test on healthy adults, based on psycholinguistic factors including semantic categories, imageability, word frequency, and word length. The CNT includes two main semantic categories (living objects and artificial objects) and various items (animals, plants, tools, vehicles, and furniture); it comprises a total of 60 items. We standardized the CNT on 221 healthy adults and investigated the clinical usefulness of the test in examining naming ability among stroke patients in Korea.

## Methods

### Stage I: Test development

#### Development of the Categorical Naming Test (CNT)

Initially, 346 words were selected from “The Frequency of Modern Korean Vocabulary” [28] according to the Institute of Language and Information Studies. The words were chosen on the basis of whether they can be drawn as pictures. In the next step, items were extracted considering their psycholinguistic properties, including word frequency, word length, imageability, and consistency of the item validation reported the evaluators. Out of all potential items, only two- or three-syllabled words were included. Moreover, words that suited the criteria of word frequency were included in the test; the criteria was as follows: i) high-frequency words should have an estimated occurrence of at least 150 times per million, and ii) low-frequency items should occur no more than 30 times per million. All included words were created as grayscale drawings. Next, the items were validated by 10 participants who were undergraduate students, receiving course credit for their participation. They did not have any knowledge about the items or the study. They evaluated the appropriateness of the pictures and reported representative words for the items to confirm validity of the drawings. Thereafter, the pictures were revised according to the feedback from these 10 evaluators who participated in the item validation. As a result of these steps for item selection, 80 items remained. These items were classified on the basis of their conceptual and semantic categories. First, they were divided into two conceptual superordinate categories, that is, concrete and abstract; then, semantic classification was carried out using semantic subordinate categories such as living objects (e.g., vegetables, fruits, flowers, mammals, fish, birds, insects, and amphibians) and artificial objects (e.g., furniture, musical instruments, transportation, writing instruments, clothing, electronics, tools, etc.), body parts, and activities/jobs [29–31]. The draft version of the CNT (80 items) comprised living objects (30 items), artificial objects (30 items), body part (5 items), and activities/jobs (15 items).

**Fig 1:**
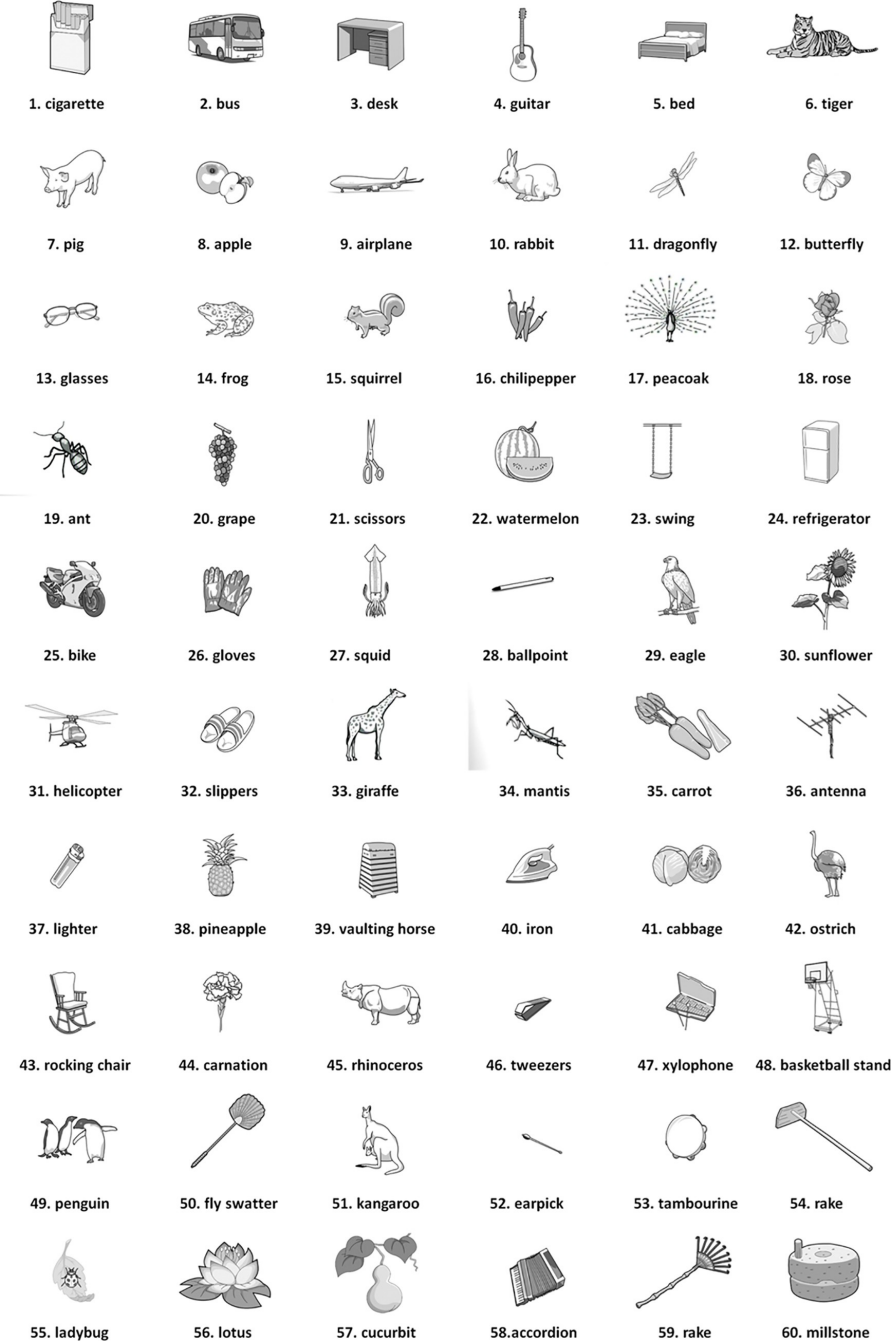
60 items of categorical naming test.

**Fig 2:**
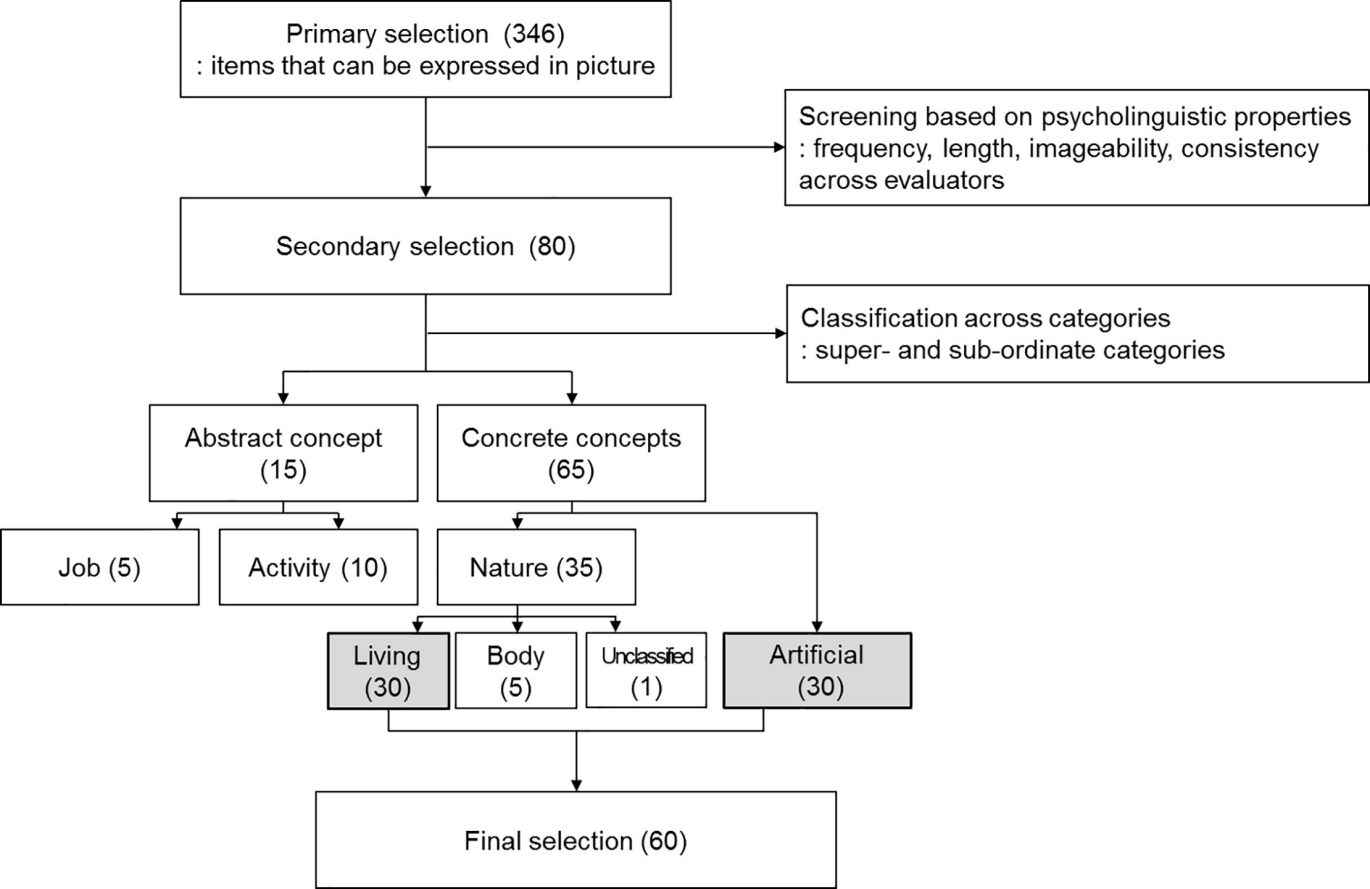
Process of item selection for the test.

For a direct comparison between the two major semantic categories of living and artificial objects, 60 items were included in the CNT in this study. The final 60 items comprised 30 words with high frequency and the remaining with low frequency (details in Figs 1 and 2).

#### Validity and reliability testing

To verify the validity of the CNT, 22 stroke patients were enrolled in the study. All the patients were diagnosed with aphasia after stroke, with an aphasia quotient (AQ) from the Korean version of the Western Aphasia Battery (K-WAB) [32], and naming scores of the K-WAB and the Korean version of the Boston Naming Test (K-BNT) [33]. The abovementioned three types of scores of the patients were used to analyze the concurrent validity of the CNT by calculating correlation coefficients among the scores.

To evaluate inter-rater reliability, two speech-language pathologists simultaneously assessed three patients with stroke. To examine test-retest reliability, the same raters administered CNT to the same patients 1~2 weeks after initial assessment. Inter-rater and test-retest reliability was determined using correlation coefficients.

### Stage II: Test standardization

#### Participants

A sample of healthy adults was recruited to evaluate the CNT for the purpose of standardization. The inclusion criteria were as follows: (1) age ≥ 45 years; (2) a score of at least 1 standard deviation below the norm score considering individual age and education on the Korean version of the Mini-Mental State Examination (K-MMSE) [34]; and (3) no history of brain injury, psychiatric illness, or other neurological illness that could affect performance on the CNT. The study recruited 221 native Korean speakers, and collected their demographic data (e.g., age, sex, education, etc.) and K-MMSE scores (Table 1). All participants were stratified by sex, age group (45–54, 55–64, 65–74, and ≥75 years), and educational level (0, 1–6, 7–9, 10–12, and ≥13 years).

**Table 1:** Comparison of variables between healthy controls (N = 221) and stroke patients (N = 112).

Variable	Healthy control (N = 221)	Stroke patients (N = 112)	P-value
Age (years)	67.75 ± 7.25	59.78 ± 13.91	<0.001**
Gender			<0.001**
Male	55	64	
Female	166	48	
Education (years)	8.81 ± 4.28	9.85 ± 4.98	0.048*
K-MMSE (30)	25.90 ± 2.54	21.03 ± 7.82	<0.001**
Time post onset (day)	-	264.71 ± 897.37	
Lesion laterality, right/left	-	58/54	
Aphasia, present/absent	-	62/50	
CNT Test			
Living objects (30)	25.51 ± 3.63	20.84 ± 8.51	<0.001**
Artificial objects (30)	25.09 ± 2.89	19.79 ± 8.23	<0.001**
Total score (60)	50.59 ± 5.97	40.63 ± 16.44	<0.001**

Note.

* indicates p<0.05

** indicates p<0.01, K-MMSE = Korean version of mini mental state examination; CNT = categorical naming test.

We obtained written informed consent from all the participants. The study protocol was approved by the Institutional Review Board of the Korea University Anam Hospital.

#### Procedure

The CNT was administered to the participants by physiatrists, speech and language pathologists, and clinical psychologists who had completed education and training for administrating and scoring of the CNT. Each participant was tested individually and was instructed to say the name of each item one by one. The examiner wrote down the participant’s responses on the answer sheet for checking phonological or semantic errors and articulatory omissions. The test had no time limit and the examiner gave participants enough time to recall and respond until they declined to answer due to a lack of knowledge. All participants were identically exposed to all 60 items.

#### Statistical analysis

Statistical analyses were performed using SPSS Statistics version 24 software (IBM, Armonk, New York, USA). A P value less than 0.05 was considered statistically significant.

Inter-item consistency was measured by Cronbach’s alpha. For assessing concurrent validity, since there is no published standardized categorical naming test in Korean, we used AQ of the K-WAB and the naming subtest scores from the K-WAB and K-BNT. A correlation analysis was also conducted between the CNT scores and the three types of scores from the aforementioned tests.

Data analysis for the healthy adults was carried out using correlation analyses between total CNT scores and age and between total CNT scores and education, using Pearson’s correlation. A two-way ANCOVA was conducted where participants’ sex was considered as a covariate to identify the differences in total CNT scores according to age and education. Post-hoc analysis of total CNT scores in healthy controls was performed according to age and education. Furthermore, a mixed ANCOVA was conducted to investigate the categorical differences between living and artificial objects according to participants’ age and education, controlling for sex as a covariate.

### Stage III: Clinical application

#### Participants

We prospectively administered the CNT and collected data from patients with stroke from the Department of Physical Medicine and Rehabilitation at the Anam Hospital, from May 2011 to April 2015. All patients were diagnosed with stroke (cerebral infarction or hemorrhage) by magnetic resonance imaging (MRI) or a CT (computed tomography) scan beforehand. Demographic (e.g., age, sex, and education) and neurological data (e.g., time passed since onset, lesion laterality, K-MMSE score, and presence of aphasia) were collected (Table 1). The presence of aphasia was determined using the result from the K-WAB, assessed by a professional speech therapist. A total of 112 patients with stroke (64 men and 48 women, mean age = 59.78) were enrolled for the clinical application procedure of the CNT. The types of stroke were evenly distributed; 58 patients (51.8%) had cerebral infarction and 54 patients (48.2%) had cerebral hemorrhage. Among the 112 patients, 44 (39.3%) had total CNT scores below 2 standard deviations from the mean. Table 1 shows the clinical characteristics of stroke patients.

We obtained written informed consent from all the participants. The study protocol was approved by the Institutional Review Board of the Korea University Anam Hospital.

#### Procedure

The same procedure was applied for administering the test to the patients, as described for the healthy controls. Patients with stroke completed the CNT with 60 items.

#### Statistical analysis

For data analysis of stroke patients, a three-way ANCOVA was conducted where age, sex, and years of education were considered as covariates in order to identify how the total scores of CNT were affected by variables such as type of stroke, lesion laterality, and presence of aphasia. Further, a mixed ANCOVA was conducted to investigate the difference in naming living versus artificial objects, with the same covariates that were used in data analysis for healthy controls. Multiple regression analysis was carried out to identify which factors showed the greatest influence in relation to the total scores obtained on the CNT.

## Results

### Results of CNT standardization

A total of 221 healthy participants (55 men, 166 women) were enrolled for the CNT standardization procedure. The mean age of the participants was 67.75 years, and the mean duration of education was 8.81 years. The mean K-MMSE score was 25.90 points (Table 1), and the mean total score on the CNT was 50.59 (out of 60 points). Pearson correlation coefficients showed a significant correlation between age, education, and total CNT scores (age, r = −.45, p <0.001; education, r = .29, p <0.001). The total CNT scores differed significantly by sex [t (219) = −2.23, p = 0.027].

The two-way ANCOVA results showed that the total CNT score was significantly different by age group and education (Table 2). Further, there was no significant interaction effect between the groups based on age and education. A post-hoc analysis of the total CNT scores showed statistically significant mean differences between the group with no education and group with certain levels of education; total CNT scores also differed between three age groups (45–64, 65–74, and ≥ 75 years). S1 Table contains the details of the total CNT scores in accordance with participants’ age and education.

**Table 2:** Two-way ANCOVA results for total categorical naming test scores in healthy controls (N = 221).

Source	Type III Sum of squares	Df	Mean Square	F	Sig.
Sex (covariate)	89.28	1	89.28	3.38	0.07
Age group (A)	559.80	3	186.60	7.07	< 0.01**
Education (E)	392.59	4	98.15	3.72	< 0.01**
A × E	383.98	11	34.91	1.32	0.21
Error	5303.03	201	26.38		
Total	573507.00	221			
Corrected Total	7829.35	220			

Note.

** indicates p<0.01.

The mean scores for naming living and artificial objects were 25.51 (out of 30) and 25.09 (out of 30), respectively. The mixed ANCOVA result revealed significant differences between the two categories [F (1, 201) = 7.70, p < 0.01] (Table 3). Additional statistical analysis of the two categories was performed according to age and education, and results showed significant differences between the two categories in the age group of 45–64 years [F (1,70) = 7.46, p < 0.01] and in the group with 1–13 years of education [F (1,203) = 4.18, p < 0.05].

**Table 3:** Mixed ANCOVA results for category effect (living objects vs. artificial objects) in healthy controls (N = 221).

Source	Type III sum of squares	Df	Mean square	F	Sig.
**Between Subjects**					
Sex (covariate)	44.64	1	44.64	3.38	0.07
Age group (A)	279.90	3	93.30	7.07	< 0.01**
Education (E)	196.29	4	49.07	3.72	< 0.01**
A × E	191.99	11	17.45	1.32	0.21
Error	2651.51	201	13.19		
**Within Subjects**					
Category (C)	27.96	1	27.96	7.70	< 0.01**
C × Sex (covariate)	55.95	1	55.95	15.40	< 0.01**
C × A	3.54	3	1.18	0.33	0.81
C × E	5.22	4	1.31	0.36	0.84
C × A × E	30.27	11	2.75	0.76	0.68
Error	730.22	201	3.63		

Note.

** indicates p<0.01.

### Internal consistency, validity, and reliability of the CNT

Internal consistency of the CNT was high, and the Cronbach’s alpha coefficient for the total number of items (60) was 0.85. Cronbach’s alpha coefficients for the two subcategories were 0.71 (living objects) and 0.69 (artificial objects).

The assessment of concurrent validity revealed significant correlations between total CNT scores and AQ of the K-WAB (r = 0.62, p <0.01), and naming scores of the K-WAB (r = 0.69, p < 0.01) and the K-BNT (r = 0.65, p < 0.01).

The correlation coefficients of inter-rater and test-retest reliabilities for the overall CNT were high (r = 0.96 and r = 0.98, respectively) but not statistically significant. For two subcategories, inter-rater and test-retest reliabilities were also high but not significant (r = 0.98 and r = 0.99 for living object and r = 0.93 and r = 0.97 for artificial objects, respectively).

### Clinical application in patients with stroke

The mean total score of the CNT for 112 patients was 40.62. S2 Table includes the details of the total CNT scores according to stroke type and lesion laterality. A three-way ANCOVA result showed no significant overall interaction according to the type of stroke, presence of aphasia, and lesion laterality [F(1,101) = 2.30, p > 0.05]. However, there was a significant difference in the total CNT scores by lesion laterality [F (1,101) = 4.16, p < 0.05]. Moreover, the total CNT scores were statistically significant according to the presence of aphasia following stroke, in these patients [F (1,101) = 16.02, p < 0.01]. Table 4 outlines the detailed values of the analysis.

**Table 4:** Three-way ANCOVA results for total categorical naming test scores in stroke patients (N = 112).

Source	Type III Sum of squares	Df	Mean square	F	Sig.
Sex (covariate)	99.30	1	99.30	0.49	0.49
Age (covariate)	0.59	1	0.59	0.00	0.96
Education (covariate)	256.03	1	256.03	1.26	0.26
Type of stroke (S)	159.89	1	159.89	0.79	0.38
Lesion side (L)	844.06	1	844.06	4.16	< 0.05*
Presence of aphasia (P)	3254.04	1	3254.04	16.02	< 0.01**
S × L	561.30	1	561.30	2.76	0.10
S × P	749.92	1	749.92	3.69	0.06
L × P	89.87	1	89.87	0.44	0.51
S × L × P	466.14	1	466.14	2.30	0.13
Error	20512.14	101	203.09		
Total	214850.00	112			
Corrected total	30006.25	111			

Note.

* indicates p<0.05

** indicates p<0.01.

A mixed ANCOVA was conducted to find differences between the CNT scores achieved according to categories of living versus artificial objects. Results showed that the categorical scores did not differ according to the type of stroke, lesion laterality, or presence of aphasia, and there was no significant interaction effect [F(1,101) = 0.001, p > 0.05] (Table 5).

**Table 5:** Mixed ANCOVA results for category effect (living objects vs. artificial objects) in stroke patients (N = 112).

Source	Type III Sum of squares	df	Mean square	F	Sig.
**Between Subjects**					
Sex (covariate)	49.65	1	49.65	0.49	0.49
Age (covariate)	0.29	1	0.29	0.00	0.96
Education (covariate)	128.02	1	128.02	1.26	0.26
Type of stroke (S)	79.95	1	79.95	0.79	0.38
Lesion side (L)	422.03	1	422.03	4.16	< 0.05*
Presence of aphasia (P)	1627.02	1	1627.02	16.02	< 0.01**
S × L	280.65	1	280.65	2.76	0.10
S × P	374.96	1	374.96	3.69	0.06
L × P	44.94	1	44.94	0.44	0.51
S × L × P	233.07	1	233.07	2.30	0.13
Error	10256.07	101	101.55		
**Within Subjects**					
Category (C)	0.04	1	0.04	0.01	0.93
C × Sex (covariate)	17.27	1	17.27	3.61	0.06
C × Age (covariate)	3.94	1	3.94	0.82	0.37
C × Education (covariate)	6.69	1	6.69	1.40	0.24
C × S	0.56	1	0.56	0.12	0.73
C × L	14.78	1	14.78	3.09	0.08
C × P	7.10	1	7.10	1.48	0.23
C × S × L	3.58	1	3.58	0.75	0.39
C × S × P	0.38	1	0.38	0.08	0.78
C × L × P	0.00	1	0.00	0.00	1.00
C × S × L × P	0.00	1	0.00	0.00	0.98
Error	483.47	101	4.79		

Note

* indicates p<0.05

** indicates p<0.01.

Multiple regression analysis was carried out to identify major attributing factors affecting the total CNT scores. The multiple regression analysis accounting for all possible predictors yielded an overall R^2^ of 0.27; the effective predictors for the CNT score were the presence of aphasia (presence or absence) (B = 12.47, β = 0.38, p < 0.01) and lesion laterality (left or right hemisphere) (B = 7.04, β = 0.21, p < 0.05) (Table 6).

**Table 6:** Multiple regression analysis summary for factors predicting categorical naming test scores in stroke patients.

	Unstandardized coefficient		Standardized coefficient	t	Sig.	F	Sig.	R^2^	adj. R^2^
	B	SE	β						
**Model**						6.57	< 0.01**	0.27	0.23
**(constant)**	7.14	13.64		0.52	0.60				
**Sex**	-1.74	3.11	-0.05	-0.56	0.58				
**Education**	.41	0.35	0.13	1.18	0.24				
**Age**	0.03	0.13	0.02	0.20	0.84				
**Stroke type (Infarction/Hemorrhage)**	0.50	3.10	0.02	0.16	0.87				
**Presence of aphasia**	12.47	3.10	0.38	4.03	< 0.01**				
**Lesion laterality (Left/Right)**	7.04	3.10	0.21	2.27	< 0.05*				

Note.

* indicates p<0.05

** indicates p<0.01.

## Discussion

We developed a new CNT for patients with stroke to reflect key features of psycholinguistic factors, such as semantic category and word frequency. The test was standardized using a sample of 221 healthy adults nationwide, with a high degree of concurrent validity for stroke patients. Administration of the CNT to 22 stroke patients with aphasia showed that the newly developed CNT has the potential to be used as a naming assessment tool for stroke patients with high validity.

Our normative data showed that the effects of word category were observed across all age and educational groups. Performance on the two main categories (living and artificial objects) showed that naming objects under the artificial category led to poor performance among all age groups (S1 Table).

These results are consistent with previous studies investigating object identification in a normal population by a speed presentation paradigm [35, 36]. According to these studies, normal subjects consistently showed poor performance when naming non-living objects compared to naming living objects. This finding may be explained by the structural differences between living and artificial objects based on visual characteristics. Living objects may have high structural similarity within their semantic category, which includes highly stable visual representations. In contrast, artificial objects exhibit low structural similarity, which is accompanied by high intra-item variation in representation. The observation that artificial objects have multiple representations in the real world may reduce their degree of familiarity in stimulus pictures used in the test, which may, in turn, cause impairment in naming and recognition in this category. This can explain the poor performance in naming non-living objects in previous studies [36–39].

Our stroke data showed that total CNT scores were significantly different according to lesion laterality and presence of aphasia, even after controlling for age, education, and sex. There was no interaction effect between lesion laterality and presence of aphasia. The group with right hemispheric lesions was superior to that with left hemispheric lesions in terms of total CNT scores. In addition, the group without aphasia was superior to the group with aphasia in terms of total CNT scores. Multiple regression results also showed that the predictors influencing the total CNT scores were related to factors such as lesion laterality and presence of aphasia, which was consistently shown with ANCOVA results in this study. These findings may be explained in terms of language lateralization in participants. Lateralization of the brain hemispheres refers to a functional dominance of one hemisphere over the other, in which one is more responsible or entirely responsible for control of a particular function in comparison to the other [40]. Language lateralization indicates a phenomenon in which one hemisphere shows greater involvement in language function than the other; this is typically the left hemisphere [41]. The CNT is fundamentally a language assessment and evaluation tool, and therefore, it is highly oriented to the functions lateralized by the left hemisphere, which is in accordance with the poor performance shown by patients with aphasia who had a left hemisphere lesion.

The results of stroke patients, however, showed no significant category differences according to the type of stroke, lesion laterality, and presence of aphasia after controlling for age, education, and sex. The fact that our data did not show category differences in naming living and artificial objects gives us some implications to consider. First, the fact that the category effect observed in normative data was not observed for all three criteria (type of stroke, lesion laterality, and presence of aphasia) indicates that the performance of the stroke patient group was indeed different from that of the heathy control group. Second, the category-specific deficit can be an individual factor of each patient, which is not determined by predictors such as stroke type, lesion laterality, and presence or absence of aphasia. In other words, it should be diagnosed by considering the individual patient’s performance profile.

In our study, healthy adults exhibited higher scores for living objects compared to artificial objects, and some patients clearly showed asymmetrical categorical naming performance on the CNT. A number of semantic memory organization theories could explain these category-specific deficits. These theories include conceptual structure, correlation between distinguishing features, domain-specific hypotheses, hierarchical interaction, organized unitary content, psychological distance, and sensory/functional theory [17, 42–48]. These theories emphasize the relative importance of several factors, including the type of knowledge, distinguishing and correlated features, visual similarity and complexity, concept similarity, concept name frequency, and domain-specific processing channels.

Word finding is considered as one of the core communication skills involving multiple stages of language processing. It encompasses a wide range of clinical phenomena and symptoms. Problems with word finding are a major obstacle interfering with patients’ self-expression. Therefore, it is imperative to investigate word-finding difficulties as language impairments. Studies have focused on different issues including spontaneous words without content or proper nouns, naming of familiar items found in pictures, verbal descriptions, word frequency, word category, speech-related errors, and cueing effects [8, 20, 49].

Above all, identifying specific word category naming deficits among patients with communication disorders after stroke is very important for devising appropriate treatment plans in treating patients with naming difficulties. In this study, we have developed for the first time, a standardized test of two representative categories (living and artificial objects) with reference to stroke patients, to determine naming difficulties based on stroke type, brain lesion location, and presence or absence of aphasia.

Our findings show that the CNT was useful in evaluating naming ability in patients with stroke. However, further studies are needed to develop and standardize extended tests under additional categories to determine the specific performance profiles in patients with different types of aphasia and other types of brain damage, which will allow for identification of the cut-off scores based on specificity and sensitivity of the data. Instead of absolute cut-off values, we provided reference tables of means and standard deviations and percentile values. These may help facilitate clinical and research use of the CNT in patients with stroke.

## Supporting information

S1 Table Categorical naming test scores according to age and education groups (N = 221). (DOCX)

S2 Table Categorical naming test scores according to stroke type and lesion laterality (N = 112). (DOCX)

S1 Dataset (ZIP)

S1 Appendix (DOCX)
