# Supplementary material for: Development of a categorical naming test in Korean: Standardization and clinical application for patients with stroke
Source: PLoS One. 2021 Feb 19;16(2):e0247118. doi: 10.1371/journal.pone.0247118 (PMC7895370; doi:10.1371/journal.pone.0247118)
Supplement: S1 Table — (DOCX) [file pone.0247118.s001.docx]

**S1 Table. Categorical naming test scores according to age and education groups (N = 221)**

|  | n | CNT Total (60)  Mean (SD) | Living objects (30)  Mean (SD) | | Artificial objects (30)  Mean (SD) | |  |  |  |
| --- | --- | --- | --- | --- | --- | --- | --- | --- | --- |
| *Age (years)* |  |  |  | | |  |  |  |  |
| 45−64 | 71 | 53.73 (4.51) | 27.20 (2.56) | | | 26.54 (2.40) |  |  |  |
| 65−74 | 110 | 50.22 (5.88) | 25.29 (3.85) | | | 24.98 (2.75) |  |  |  |
| 75 ≥ | 40 | 46.05 (5.28) | 23.10 (3.16) | | | 22.95 (2.69) |  |  |  |
|  |  |  |  | | |  |  |  |  |
| *Education (years)* |  |  |  | | |  |  |  |  |
| 0 | 17 | 45.41 (5.94) | 23.12 (3.77) | | | 22.29 (2.97) |  |  |  |
| 1−13 ≥ | 204 | 51.02(5.78) | 25.71 (3.56) | | | 25.32 (2.77) |  |  |  |
|  |  |  |  |  | | | | |  |
| Total | 221 | 50.59 (5.97) | 25.51 (3.63) | | | 25.09 (2.89) |  |  |  |
