# Supplementary material for: Development of a categorical naming test in Korean: Standardization and clinical application for patients with stroke
Source: PLoS One. 2021 Feb 19;16(2):e0247118. doi: 10.1371/journal.pone.0247118 (PMC7895370; doi:10.1371/journal.pone.0247118)
Supplement: S2 Table — (DOCX) [file pone.0247118.s002.docx]

**S2 Table. Categorical naming test scores according to stroke type and lesion laterality (N=112)**

|  | N | CNT  Total (60)  Mean (SD) | Living objects  (30)  Mean (SD) | Artificial objects (30)  Mean (SD) |  |
| --- | --- | --- | --- | --- | --- |
|  |  |  |  |  |  |
| *Stroke Type* |  |  |  |  |  |
| Infarction | 58 | 39.90 (16.75) | 20.41 (8.70) | 19.48 (8.28) |  |
| Hemorrhage | 54 | 41.41 (16.22) | 21.30 (8.36) | 20.11 (8.21) |  |
|  |  |  |  |  |  |
| *Lesion Location* |  |  |  |  |  |
| Left hemisphere | 62 | 34.90(17.99) | 18.15 (9.39) | 16.76 (8.90) |  |
| Right hemisphere | 50 | 47.72(10.80) | 24.18 (5.82) | 23.54 (5.32) |  |
|  |  |  |  |  |  |
| *Presence of aphasia* |  |  |  |  |  |
| Present | 50 | 32.42(18.02) | 16.60(9.54) | 15.82(8.81) |  |
| Absent | 62 | 47.24(11.46) | 24.26(5.67) | 22.98(6.10) |  |
|  |  |  |  |  |  |
| Total | 112 | 40.62 (16.44) | 20.84(8.51) | 19.79 (8.22) |  |
|  |  |  |  |  |  |
