# Supplementary material for: Development of a categorical naming test in Korean: Standardization and clinical application for patients with stroke
Source: PLoS One. 2021 Feb 19;16(2):e0247118. doi: 10.1371/journal.pone.0247118 (PMC7895370; doi:10.1371/journal.pone.0247118)
Supplement: S1 Appendix — (DOCX) [file pone.0247118.s004.docx]

**Appendix**

|  | | **K-CNT total (60)** | | | **Living objects (30)** | | | **Artificial objects (30)** | | |
| --- | --- | --- | --- | --- | --- | --- | --- | --- | --- | --- |
| **Age (years)** | | 45−64 | 65−74 | ≥75 | 45−64 | 65−74 | ≥75 | 45−64 | 65−74 | ≥75 |
| **N** | | 71 | 110 | 40 | 71 | 110 | 40 | 71 | 110 | 40 |
| **Median** | | 55 | 52 | 46 | 28 | 27 | 24 | 27 | 25 | 23 |
| **Percentile** | **1** | 60 | 60 | 58 | 30 | 30 | 30 | 30 | 30 | 28 |
|  | **5** | 59 | 57 | 57 | 30 | 30 | 30 | 30 | 29 | 28 |
|  | **10** | 59 | 57 | 53 | 30 | 29 | 27 | 29 | 29 | 26 |
|  | **15** | 58 | 56 | 52 | 30 | 29 | 27 | 29 | 28 | 26 |
|  | **20** | 57 | 55 | 51 | 30 | 28 | 26 | 29 | 28 | 25 |
|  | **25** | 57 | 54 | 49 | 29 | 28 | 25 | 28 | 27 | 25 |
|  | **30** | 57 | 54 | 48 | 29 | 28 | 25 | 28 | 27 | 24 |
|  | **35** | 57 | 53 | 48 | 29 | 27 | 24 | 28 | 26 | 24 |
|  | **40** | 56 | 53 | 48 | 28 | 27 | 24 | 27 | 26 | 23 |
|  | **45** | 55 | 52 | 47 | 28 | 27 | 24 | 27 | 25 | 23 |
|  | **50** | 55 | 52 | 46 | 28 | 27 | 24 | 27 | 25 | 23 |
|  | **55** | 54 | 51 | 45 | 28 | 26 | 23 | 27 | 25 | 23 |
|  | **60** | 54 | 50 | 45 | 27 | 25 | 22 | 26 | 24 | 23 |
|  | **65** | 53 | 49 | 44 | 26 | 25 | 21 | 26 | 24 | 22 |
|  | **70** | 52 | 48 | 43 | 26 | 24 | 21 | 26 | 23 | 22 |
|  | **75** | 51 | 47 | 43 | 26 | 23 | 20 | 25 | 23 | 22 |
|  | **80** | 50 | 45 | 42 | 25 | 22 | 20 | 24 | 23 | 21 |
|  | **85** | 49 | 45 | 41 | 24 | 22 | 19 | 24 | 22 | 21 |
|  | **90** | 47 | 42 | 38 | 24 | 19 | 19 | 23 | 21 | 20 |
|  | **95** | 44 | 37 | 36 | 22 | 18 | 18 | 22 | 20 | 17 |
